# Prelimbic Ensembles Mediate Cocaine Seeking After Behavioral Acquisition and Once Rats Are Well-Trained

**DOI:** 10.3389/fnbeh.2022.920667

**Published:** 2022-09-26

**Authors:** Bo W. Sortman, Christina Gobin, Samantha Rakela, Berk Cerci, Brandon L. Warren

**Affiliations:** Warren Lab, Department of Pharmacodynamics, University of Florida, Gainesville, FL, United States

**Keywords:** prefrontal cortex, addiction, learning and memory, engram, acquisition

## Abstract

Substance use disorder (SUD) is a chronic relapsing condition characterized by continued use of drugs despite negative consequences. SUD is thought to involve disordered learning and memory wherein drug-paired cues gain increased salience, and ultimately drive craving and relapse. These types of associations are thought to be encoded within sparsely distributed sets of neurons, called neuronal ensembles, that drive encoded behaviors through synchronous activity of the participant neurons. We have previously found that Fos-expressing neuronal ensembles within the prefrontal cortex are required for well-trained cocaine seeking. However, less is known about how quickly cortical neuronal ensembles form during the initiation of cocaine seeking behavior. Here, we seek to further elucidate the role of Fos-expressing neuronal ensembles within the prelimbic cortex (PL) after the initial acquisition of cocaine self-administration (SA), or, after 10 days of additional SA training (well-trained). We trained *Fos-LacZ* transgenic rats to lever press for cocaine under an FR1 schedule of reinforcement. Once rats met acquisition criteria for cocaine self-administration, we ablated Fos-expressing neuronal ensembles in the PL using the Daun02 inactivation method, either 1 or 10 days after the rats met the acquisition criteria. Targeted ablation of Fos-expressing neuronal ensembles in the PL attenuated active lever pressing both 1 day and 10 days after rats acquired cocaine self-administration. Together, this suggests that Fos-expressing neuronal ensembles rapidly form in the PL and continue to mediate maintained cocaine seeking behavior.

## Introduction

Substance use disorders, including cocaine use disorder (CUD), involve associative learning systems. Cocaine use can increase the salience of cocaine-paired cues that can drive continued use, craving, and relapse (Robinson and Berridge, 1993; Hyman, 2005). Cocaine-associated cues elicit strong physiological arousal and increased craving in individuals with CUD (Childress et al., 1988). Cue-induced craving can lead to relapse in individuals even after prolonged abstinence (O’Brien et al., 1992; Lu et al., 2004). In animal models, cocaine-associated cues induce a robust reinstatement of cocaine seeking behavior (Weiss et al., 2000; Capriles et al., 2003; Fuchs et al., 2004). Additionally, the strength of cocaine craving after the initial cocaine experience is a strong predictor of CUD (Lambert et al., 2006). The associations between cue and reinforcer, and the neurological mechanisms underpinning these associations are particularly important for a broader understanding of the etiology of CUD.

Neuronal ensembles contain sparsely distributed, interconnected neurons that are thought to encode learned associations (Hebb, 1949). Testing this hypothesis has become possible with the development of novel methods targeting neurons that express immediate early genes, like *c-Fos* and its protein product Fos. Fos is expressed after strong neural activity (Cruz et al., 2013), making it an ideal proxy marker for strongly activated ensemble neurons. Neuronal ensembles across distinct brain regions are critical for drug seeking behavior (Bossert et al., 2011; Cruz et al., 2014). Our lab has previously found that neuronal ensembles in the infralimbic (IL) region of the medial prefrontal cortex (mPFC) mediate self-administration and extinction of food, cocaine, and oxycodone self-administration (Warren et al., 2016, 2019; Kane et al., 2021; Gobin et al., 2022). Furthermore, ensemble-specific manipulations also alter behavioral responses in multiple memory-dependent tasks (Cruz et al., 2013; Suto et al., 2016; Warren et al., 2017; Frankland et al., 2019; Vetere et al., 2019; Josselyn and Tonegawa, 2020).

The hippocampus is widely regarded to be the structure primarily responsible for encoding initial learned experiences; driven through rapid synaptic plasticity following exposure to novel stimuli (Eagle et al., 2016). Information from the hippocampus is thought to be subsequently directed to the cortex (McClelland et al., 1995; O’Reilly and Rudy, 2001; Frankland and Bontempi, 2005; Winocur et al., 2010). However, recent evidence has emerged that the PL rapidly recruits neuronal ensembles that are responsive to novel stimuli (Takehara-Nishiuchi et al., 2020). This suggests that learned experiences may be consolidated in the cortex concomitantly with hippocampal processing, rather than initially stored in the hippocampus and subsequently moved to the cortex. Additionally, PL ensembles are highly specific to behavior soon after experience and adapt over the learning process (Zhang et al., 2021). However, it is currently unknown how early in the learning process Fos-expressing PL ensembles become behaviorally relevant in cocaine seeking. Therefore, we intend to advance this literature by testing the role of Fos-expressing neuronal ensembles in the prelimbic (PL) region of the mPFC in cocaine seeking after behavioral acquisition. Here we seek to test two hypotheses: (1) that Fos-expressing neuronal ensembles mediate recently acquired cocaine self-administration (SA) and; (2) that Fos-expressing PL neuronal ensembles mediate cocaine SA once rats are well-trained.

To test these hypotheses, we trained rats to lever press for intravenous infusions of cocaine. Next, we inactivated Fos-expressing neuronal ensembles either 24 h after behavioral acquisition or 10 days after acquisition by microinfusing Daun02 into the PL in *Fos-LacZ* transgenic rats. *Fos-LacZ* rats express β-galactosidase (β-gal) under the control of the Fos promoter. Daun02 is a prodrug that interacts with β-gal to produce the apoptosis-promoting molecule daunorubicin, resulting in the ablation of neurons expressing Fos-promoter driven β-gal. This procedure, therefore, allows targeted ablation of recently activated, and consequently Fos-expressing neurons (Koya et al., 2009a; Cruz et al., 2013). We then measured their cocaine seeking behavior after inactivating the acquisition or well-trained cocaine seeking ensemble.

## Methods

### Subjects

We used adult male (*n* = 42) and female (*n* = 25) *Fos-lacZ* transgenic rats (*N* = 67), weighing 171–458 g at the beginning of the experiment. We single-housed rats under a reverse 12 h light/dark cycle (lights off at 10:00 a.m.). Food was restricted to 20 g of chow per day, with *ad libitum* access to water. All animal procedures were approved by the relevant Institutional Animal Care and Use Committee. We excluded 17 rats from all analyses: nine failed to display cocaine seeking behavior on induction day and eight had misplaced cannula. One rat was excluded from β-gal analysis in Experiment 2 due to poor labeling but was included in the behavioral analysis.

### Surgery

We deeply anesthetized rats with isoflurane (5% induction, 3% maintenance) during intracranial cannula and intravenous jugular catheter implantation. We administered buprenorphine (0.03 mg/kg, s.c.) and meloxicam (5 mg/kg, s.c.) daily for 3 days after surgery for analgesia. We allowed rats 4 days to recover before starting self-administration training.

#### Intracranial Cannula Implantation

We implanted permanent guide cannulas (23-gauge, Plastics One) bilaterally 1 mm above the PL. The nose bar was set at −3.3 mm, and the coordinates used for the PL were anteroposterior: 3.0, mediolateral: 1.5, and dorsoventral: −2.8 (10° angle). We used dental cement and jeweler’s screws to adhere cannulas to the rat’s skull.

#### Intravenous Catheter Implantation

We implanted SILASTIC catheters into the jugular vein as described previously (Warren et al., 2019; Kane et al., 2021). We constructed catheters such that 3 cm of tubing entered the jugular vein, while the other end of the tubing was affixed to a modified 22-gauge cannula adhered to a custom-made backmount. We threaded the proximal end of the tubing subcutaneously between the shoulder blades and ported the modified cannula through the midscapular region of the back. We flushed catheters daily with 0.2 ml gentamicin in sterile saline (4.25 mg/ml; APP Pharmaceuticals).

### Daun02 Inactivation Method and Cocaine Self-administration

#### Intracranial Infusions

On induction day, we bilaterally microinfused (2 μg/0.5 μl/side) of Daun02 or sterile 1× PBS into the PL over a 1 min period using 10 μl Hamilton syringes and a syringe pump (Kent Scientific, Torrington, CT, USA) with polyethylene-50 tubing to 30-gauge injectors (RWD Life Science, Guangdong, China) that extended 1 mm below the guide cannula. Injectors were left in the cannula for 1 min after infusion before being removed.

#### Drugs

We used cocaine hydrochloride acquired from NIDA Drug Supply Program. We selected an intravenous dose of 0.75 mg/kg/infusion with a volume of 0.1 ml per infusion for self-administration, dissolved in sterile saline, based on previous experiments (Ward et al., 2005). We obtained Daun02 from Medchem Express and dissolved it in a vehicle containing 5% DMSO, 6% Tween 80, and 89% 0.01 M PBS. We chose the dose of Daun02 based on previous studies (Koya et al., 2009b; Bossert et al., 2011; Cruz et al., 2014).

#### Apparatus

We trained, and tested rats in Med Associates self-administration chambers. Each chamber contained a house light and fan that remained on for the duration of each session. Each chamber was housed in a sound attenuating box and contained an active and inactive lever. Above each lever was a light and a speaker. Pressing the active lever resulted in activation of the cue light directly above the active lever, initiation of the tone cue and delivery of a 0.75 mg/kg/infusion of cocaine, with a 10 s timeout during which active lever presses did not result in additional infusions. Pressing the inactive lever had no programmed consequences.

#### Self-administration Training for Cocaine and Behavioral Acquisition

We placed rats into operant chambers (described above) for 3 h per day and trained them to press an active lever which resulted in a light/sound cue and 0.75 mg/kg infusion of cocaine on an FR1 schedule. We allowed rats between 1 and 12 days to reach the acquisition criteria defined as ≥30 active lever presses and ≥70% of total lever responses on the active lever. We chose these criteria based on previous studies that used similar acquisition criteria (Mitchell et al., 2005; Taylor et al., 2010; Quintana-Feliciano et al., 2021).

#### Induction Session

The purpose of the induction session is to elicit Fos and β-gal expression in neurons relevant to cocaine seeking behavior. This session is 30 min long, during which an active lever response results in identical activation of the sound and light cues, but not an infusion of cocaine. Induction sessions are performed under non-reinforced conditions to avoid potential pharmacological effects of cocaine. We chose a session duration of 30 min to minimize extinction learning. After this 30 min non-reinforced cocaine seeking test, we left rats in the operant boxes for an additional 60 min to allow for maximal β-gal expression before either microinfusing Daun02 (Experiments 2 and 3) or sacrificing rats for subsequent β-gal quantification (Experiment 1). The induction session took place either 1 day (Experiment 1 and 2) or 10 days (Experiment 3) after self-administration acquisition.

#### Test Session

The purpose of the test session was to measure the effect of ensemble ablation on cocaine seeking. The test session was 30 min long and active lever presses were not reinforced but did activate the cocaine-paired sound and light cues. Test sessions for Experiments 2 and 3 occurred 48 h after the induction session (Kane et al., 2021). After the 30 min non-reinforcing cocaine seeking test, we left rats in the operant boxes for 60 min to allow for maximal β-gal expression and sacrificed them for subsequent β-gal quantification.

#### X-Gal Histochemistry for β-Gal Quantification in *Fos-LacZ* Rats

Ninety min after the start of the induction session (Experiment 1) or test session (Experiments 2 and 3), we anesthetized rats with isoflurane and transcardially perfused rats with 1× PBS, then with 4% paraformaldehyde to fix the tissue. We extracted brains and post-fixed them in 4% paraformaldehyde for 2 h, cryopreserved them in 30% sucrose in 1× PBS until all brains had been fully saturated at 4°C, froze them on dry ice, and finally stored them at −80°C. We sectioned brains at 40 μm and collected PL sections for subsequent X-gal staining. We washed free-floating slices in 1× PBS 3× , for 10 min per wash, then incubated them in reaction buffer (0.1 M X-gal, 100 mM sodium phosphate, 100 mM sodium chloride, 5 mM EGTA, 2 mM MgCl_2_, 0.2% Triton X-100, 0.05 M K_3_Fe(CN)_6_, and 0.05 M K_4_Fe(CN)_6_) for 45 min at 37°C with gentle shaking. We washed sections in 1× PBS 3× , for 10 min per wash, mounted free-floating slides on Polysine precleaned microscope slides (25 mm × 75 mm × 1.0 mm; Thermo Scientific), and allowed them to air dry overnight. The following day, we dehydrated the slides using 30%, 60%, 90%, 95%, and 100% ethanol for 2 min each and subsequently cleared the tissue with Citrasolv (Fisher Scientific, NH, USA), and cover slipped slides with Permount (Sigma Aldrich, MO, USA).

β-gal images were captured using a Keyence BZ-X810 microscope at 200× magnification with BZ-X Analyzer software. Observers blind to the test conditions (interrater reliability: Pearson’s *r* = 0.86) used ImageJ to manually quantify β-gal expression in the PL. Three randomly selected coronal sections were quantified per rat. We then averaged the data from each rat counted as *n* = 1.

### Statistical Analysis

We analyzed all data using GraphPad Prism (version 9.1.2) software, with α set at 0.05 for all analyses performed. We used Student’s *t*-tests, Welch’s *t*-tests, paired *t*-tests, or two-way ANOVA to analyze behavioral data and β-gal data, when appropriate. No sex differences were detected on test-day lever pressing in any experiment (*p* > 0.05), therefore data from males and females were combined.

#### Experimental Design and Statistical Analysis

##### Experiment 1: PL β-Galactosidase Expression After Acquisition of Cocaine Seeking

The purpose of Experiment 1 was to measure PL β-gal expression in home cage controls (No test) and rats that underwent a non-reinforced cocaine seeking session (Test) after meeting acquisition criteria. We hypothesized that rats in the Test group would recall cocaine self-administration and exhibit cocaine seeking behavior, thereby reactivating the cocaine seeking neuronal ensemble. We predicted that this neuronal activation would drive the expression of Fos and β-gal, resulting in greater β-gal expression in Test vs. No test rats. Experiment 1 was a single-factor between-subjects design with two groups (Test vs. No test). We trained rats to self-administer cocaine (0.75 mg/kg/inf) under an FR1 schedule of reinforcement for up to 12 days until each individual rat met behavioral acquisition criteria. Twenty-four hours after the session of behavioral acquisition, we randomly assigned rats to euthanasia directly from their home cage (No test) or subjected them to a 30 min non-reinforced induction session (Test). We then euthanized test rats 90 min after the start of the induction session. All tissue harvested was then used for β-gal staining and quantification.

##### Experiment 2: Daun02 Inactivation of the Fos-Expressing PL Neuronal Ensemble After Acquisition of Cocaine Seeking

The purpose of Experiment 2 was to test the hypothesis that a PL neuronal ensemble mediates cocaine self-administration after initial behavioral acquisition. Experiment 2 was a single-factor between-subjects design with two groups (Vehicle vs. Daun02). To test this hypothesis, we allowed rats up to 12 days to meet behavioral acquisition criteria. Twenty-four hours after each rat met acquisition criteria, we assigned rats to either the Vehicle or Daun02 group based on their active lever responses during the acquisition session in which the rat met acquisition criteria. We then allowed rats to lever press for cocaine-paired cues in a 30 min non-reinforced induction session intended to drive β-gal expression in neurons associated with cocaine seeking behavior. Ninety minutes after the start of the induction session, we microinfused either Vehicle or Daun02 into the PL. Forty-eight hours after the induction test (to allow for apoptosis), we returned rats to the operant chamber for a final 30 min non-reinforcing cocaine seeking test. Ninety minutes after the start of this test, we euthanized rats and harvested their brains for subsequent β-gal analysis.

##### Experiment 3: Daun02 Inactivation of Fos-Expressing PL Neuronal Ensembles After Well-Trained Cocaine Seeking

The purpose of Experiment 3 was to test the hypothesis that a PL neuronal ensemble mediates cocaine self-administration in well-trained rats. Experiment 3 was a single-factor between-subjects design with two groups (Vehicle vs. Daun02). To test this hypothesis, we allowed rats up to 12 days to meet behavioral acquisition criteria. Once individual rats met the acquisition criteria for cocaine SA, we trained them for 10 additional days. Twenty-four hours after their 10th day of training post-acquisition we assigned rats to either the Vehicle or Daun02 group based on their active lever responses during their final training session. We then allowed rats to lever press for cocaine-paired cues in a 30 min non-reinforced induction session, to drive β-gal expression in neurons associated with cocaine seeking behavior. Ninety minutes after the start of the induction test, we microinfused either Vehicle or Daun02 into the PL. Forty-eight hours after the induction session, we returned rats to the operant chamber for a final 30 min non-reinforcing cocaine seeking test. Ninety minutes after the start of this final test, we euthanized rats and harvested their brains for subsequent β-gal analysis.

## Results

### Experiment 1: Measure β-Galactosidase Expression in Rats Exposed to the Context Associated With Behavioral Acquisition

**Figure 1:**
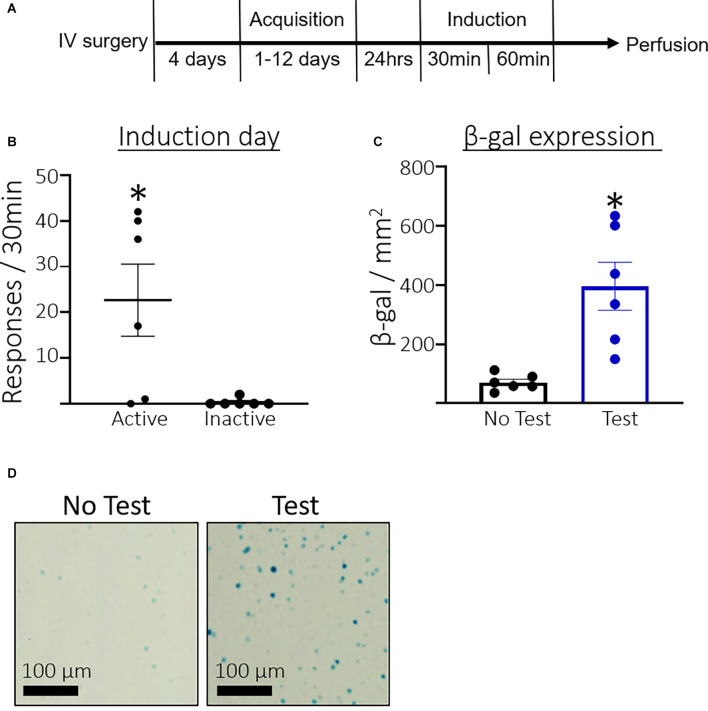
Experiment 1: Prelimbic cortex (PL) β-galactosidase expression after acquisition of cocaine seeking. **(A)** Timeline showing the behavioral procedure. We implanted jugular catheters and allowed 4 days for rats to recover. We trained rats to lever press for infusions of cocaine in 3 h daily sessions for between 1 and 12 days until they reached acquisition criteria of ≥30 active lever presses and ≥70% of total lever responses on the active lever. We tested rats 24 h after acquisition in a 30 min non-reinforcing cocaine seeking induction session. Rats were sacrificed 60 min after the session ended and euthanized for β-gal quantification. **(B)** The number of total active and inactive lever responses in the induction session. **(C)** The number of β-gal positive nuclei per mm^2^ in the PL. **(D)** Representative images showing β-gal labeling in the PL. Data are presented as mean ± SEM (*n* = 6 per group). *p < 0.05 compared to home cage controls.

Figure 1A shows the timeline for Experiment 1. Rats took an average of 4.33 days (SEM = 0.45, *n* = 12) to reach the acquisition criteria. Figure 1B shows the total number of active and inactive lever responses during the induction session for rats that were assigned to the Test condition. A paired *t*-test found that rats pressed significantly more on the active compared to the inactive lever (*t*_(5)_ = 2.8, *p* = 0.0375) during the induction session. Figure 1C shows the total number of β-gal positive nuclei per mm^2^. Rats in the Test condition expressed significantly more β-galactosidase than the No test controls (*t*_(5.185)_ = 3.970, *p* = 0.0099). Additionally, we found a strong correlation between active lever pressing and β-galactosidase expression in the test condition, *r*_(4)_ = 0.79 *p* = 0.058. Figure 1D shows representative β-galactosidase expression.

### Experiment 2: Determine the Role of the Fos-Expressing PL Ensemble After Acquisition of Cocaine Seeking

**Figure 2:**
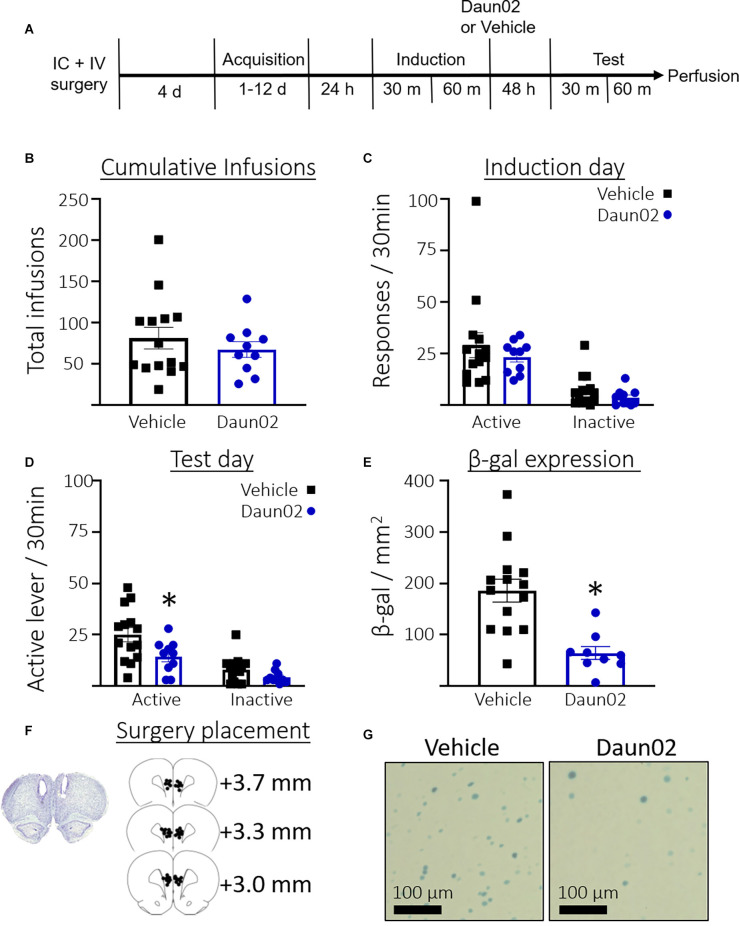
Experiment 2: Role of PL ensembles after acquisition of cocaine seeking. **(A)** Timeline showing the behavioral procedure. **(B)** The number of total infusions earned across all acquisition days between rats assigned to either the vehicle or Daun02 condition. **(C)** The number of active and inactive lever responses in the induction session between rats assigned to either the vehicle or Daun02 condition. **(D)** Number of active and inactive lever responses in the test session between rats that received either vehicle or Daun02. **(E)** Number of β-gal positive nuclei per mm^2^ in the PL between rats that received either vehicle or Daun02, with representative images of β-gal expression. **(F)** Cannula placement. **(G)** Representative images showing β-gal labeling in the PL. Data are presented as mean ± SEM (*n* = 14–10 per group). *p < 0.05 compared to vehicle-treated controls.

Figure 2A shows the timeline for Experiment 2. Rats took an average of 4.54 days (SEM = 0.73, *n* = 24) to reach the acquisition criteria. Figure 2B shows the total number of cocaine infusions earned over the acquisition training days in rats that would go on to receive either vehicle or Daun02. Welch’s *t*-test revealed no significant difference between the groups in total cocaine infusions received during self-administration training (*t*_(21.61)_ = 0.85, *p* = 0.40). Figure 2C shows the total responses during the induction session. Welch’s *t*-tests revealed no significant difference between the groups in total active lever presses (*t*_(16.93)_ = 0.8, *p* = 0.39) or total inactive lever presses (*t*_(20.61)_ = 1.5, *p* = 0.16). Figure 2D shows the total responses during the test session. Welch’s *t*-test revealed significantly fewer active lever presses (*t*_(21.60)_ = 2.5, *p* = 0.02), but not inactive lever presses (*t*_(19.76)_ = 1.9, *p* = 0.07) in the Daun02 condition compared to vehicle. Figure 2E shows the total β-gal expression per mm^2^ in the PL on test day. A Welch’s *t*-test revealed a significant reduction in β-gal expression in the Daun02 condition compared to the vehicle condition (*t*_(19.44)_ = 4.8, *p* = 0.0001). Figure 2F shows surgery placement and Figure 2G shows representative β-galactosidase expression.

### Experiment 3: Determine the Role of the Fos-Expressing PL Ensemble in Well-Trained Cocaine Seeking

**Figure 3:**
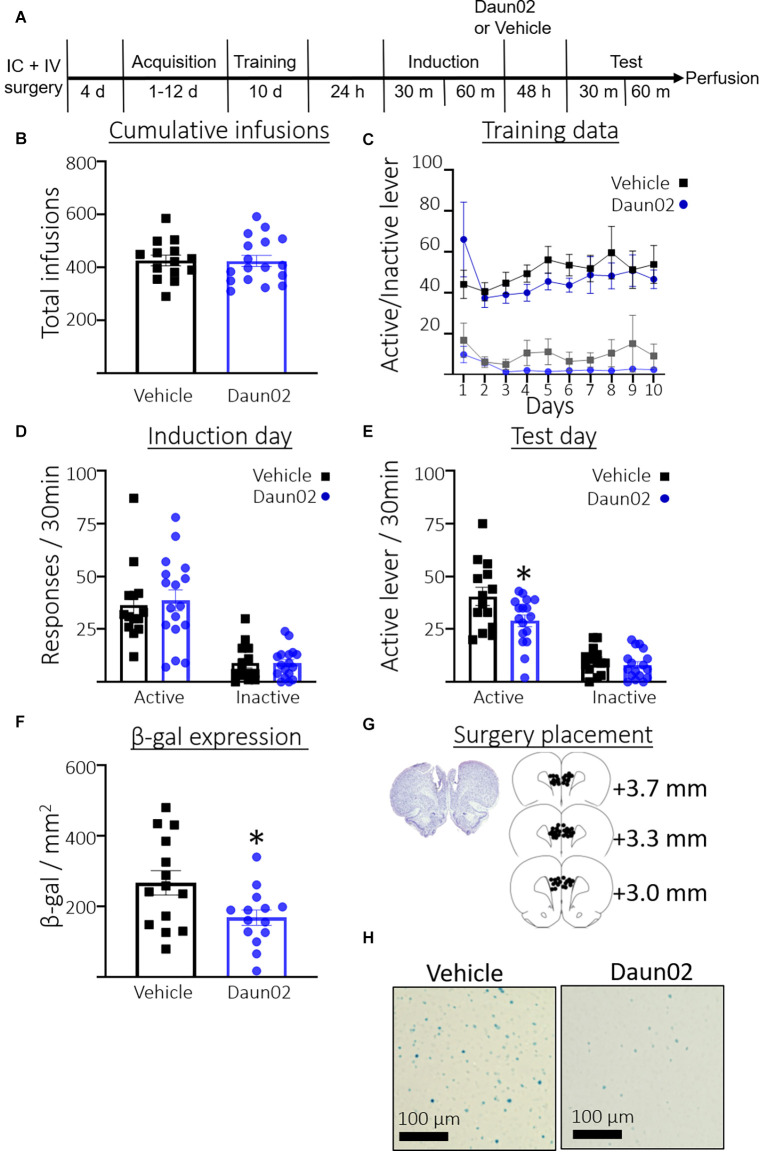
Experiment 3: Role of PL ensembles in well-trained cocaine seeking. **(A)** Timeline showing the behavioral procedure. **(B)** The number of total infusions earned across all acquisition days and subsequent training days between rats assigned to the vehicle or Daun02 condition. **(C)** Active and inactive lever responses in rats assigned to the vehicle or Daun02 condition across days of training post acquisition. **(D)** The number of active and inactive lever responses in the induction session in rats assigned to the vehicle or Daun02 condition. **(E)** The number of active and inactive lever responses in the test session between rats that received either vehicle or Daun02. **(F)** The number of β-gal positive nuclei per mm^2^ in the PL between rats that received either vehicle or Daun02. **(G)** Cannula placement. **(H)** Representative images showing β-gal labeling in the PL. Data are presented as mean ± SEM (*n* = 14–17 per group). *p < 0.05 compared to vehicle-treated controls.

Figure 3A shows the timeline for Experiment 3. Rats took an average of 4.23 days (SEM = 0.37, *n* = 31) to reach the acquisition criteria. Figure 3B shows the total number of cocaine infusions earned during acquisition training and the 10 subsequent training days post acquisition. A Student’s *t*-test revealed no significant difference between rats in the vehicle condition or Daun02 condition in total infusions earned (*t*_(28.90)_ = 0.07, *p* = 0.94). Figure 3C shows total active and inactive lever responses across the 10 training days following the acquisition session. A two-way repeated measures ANOVA (Day × Group) revealed no interaction between Day and Group (*F*_(9,261)_ = 0.96, *p* = 0.47), no main effect of Day (*F*_(3.307,95.90)_ = 1.074, *p* = 0.37), and no main effect of Group (*F*_(1,29)_ = 0.47, *p* = 0.50) on active lever responding. A two-way repeated measures ANOVA (Day × Group) revealed no interaction between Day and Group (*F*_(9,261)_ = 0.54, *p* = 0.84), no main effect of Day (*F*_1.899,55.06)_ = 1.5, *p* = 0.24), and no main effect of Group (*F*_(1,29)_ = 2.1, *p* = 0.16), on inactive lever responding. Figure 3D shows the total responses during the induction session. Welch’s *t*-test revealed no significant difference between the groups in total active lever presses (*t*_(28.84)_ = 0.33, *p* = 0.74), or inactive lever presses (*t*_(25.10)_ = 0.02, *p* = 0.98). Figure 3E shows total responses during the test session. Welch’s *t*-test revealed significantly fewer active lever presses in the Daun02 condition compared to the vehicle condition (*t*_(22.85)_ = 2.3, *p* = 0.03) Welch’s *t*-test revealed no significant difference between the groups in total inactive lever presses (*t*_(28.26)_ = 0.86, *p* = 0.40). Figure 3F shows total β-gal expression in the PL on test day. Welch’s *t*-test revealed a significant reduction in β-gal expression in the Daun02 condition compared to the vehicle condition (*t*_(21.89)_ = 2.4, *p* = 0.02). Figure 3G shows surgery placement and Figure 3H shows representative β-galactosidase expression.

## Discussion

### Fos-Expressing Neuronal Ensembles in the Prelimbic Cortex Mediate Recently Acquired and Well-Trained Cocaine Self-administration

In this study, we combined a novel behavioral acquisition procedure with the Daun02 chemogenetic inactivation procedure to test the role of PL Fos-expressing neuronal ensembles in initial and well-trained cocaine seeking behavior. We gave rats up to 12 days of cocaine SA to meet our acquisition criteria. Either 24 h or 10 days after individual rats met acquisition criteria, we assessed rats’ cocaine seeking under non-reinforced conditions, followed by intracranial infusions of Daun02. This non-reinforced seeking session was intended to reactivate neuronal ensembles associated with cocaine seeking, leading to the expression of the β-gal enzyme in these neurons. Daun02 is converted into daunorubicin within neurons expressing β-gal, triggering selective apoptosis of previously activated neurons and ablation of the Fos-expressing neuronal ensembles (Koya et al., 2009b). Two days later, we measured rats’ recall of cocaine seeking behavior in an identical non-reinforced test session. Rats that received Daun02 pressed the cocaine-paired lever significantly less than rats that received the vehicle in both the acquisition and well-trained experiments. This finding supports our hypothesis that a Fos-expressing neuronal ensemble in the PL is necessary for memory retrieval in cocaine SA. These results are congruent with our previous findings that ensembles in the mPFC modulate cocaine SA (Warren et al., 2019; Kane et al., 2021). Furthermore, these findings suggest that Fos-expressing neuronal ensembles within the PL are critical for mediating reward seeking, even after a single day of motivated self-administration (Quintana-Feliciano et al., 2021).

### Neuronal Ensembles in the PL Mediate Operant and Pavlovian Reward Responding

Neuronal ensembles are thought to develop from synchronous activation of neurons during an experiential event. After repeated pairings, the ensemble mediates learned behaviors. However, it is unknown how early ensembles become behaviorally relevant in cocaine SA. In a recent study using a Pavlovian learning paradigm (i.e., a sucrose reinforcer was paired with a sound cue) a subset of the initially active neurons was re-activated during subsequent memory recall (Brebner et al., 2019). Furthermore, persistent chemogenetic excitation of the initial ensemble during the conditioning phase reduced the ability of the mice to perform in a cue-discrimination task. This study demonstrates that neuronal ensembles are likely reactivated and refined with additional training. In agreement with the above study, experiments using two-photon calcium imaging show that not only is the initial ensemble repeatedly activated during learning, but distinct subsets of neurons encode specific aspects of the behavior (Grant et al., 2021). Furthermore, using *in vivo* single-unit recording, Takehara-Nishiuchi et al. (2020) demonstrated that PL neural activity is modulated by a novel stimulus, such that neurons that initially respond to a new experience become hyper- or hyporesponders within minutes of stimulus exposure. In line with this data, dorsal-medial prefrontal cortex (dmPFC) ensemble efferents differentially respond to a conditioned reward after learning (Otis et al., 2017). Ensembles in the dmPFC that project to the Nucleus Accumbens (NAc) become excited while ensembles that project to the paraventricular nucleus of the thalamus are inhibited during cue presentation. This adaptive response guides the initial acquisition and subsequent expression of reward seeking. Here, our Daun02 ablation occurs after the rats have acquired cocaine seeking behavior, suggesting we might be targeting and disrupting Fos-expressing PL neurons that project to the NAc. This is also in line with studies showing higher Fos activity in NAc projecting PL neurons during cue-induced reinstatement of cocaine seeking (McGlinchey et al., 2016; James et al., 2017).

We have also shown that the mPFC more broadly houses ensembles that mediate recently acquired goal-oriented responding. For instance, using a similar acquisition paradigm we have found that the infralimbic cortex (IL) is necessary for initial food and oxycodone seeking (Quintana-Feliciano et al., 2021; Gobin et al., 2022). Our current findings expand this literature and support the hypothesis that ensembles in the mPFC and specifically in the PL are formed and become behaviorally relevant early in the learning process.

### The PL Is Necessary for Drug-Related Learning and Behavior

The role of the mPFC in drug seeking has been extensively studied. Lesioning the mPFC inhibits the acquisition of cocaine-induced conditioned place preference and context-dependent behavioral sensitization (Tzschentke and Schmidt, 1998a, b, Tzschentke and Schmidt, 1999). Additionally, manipulating perineuronal nets in the PL result in the attenuation of cocaine CPP (Slaker et al., 2015). Region-wide lesion and pharmacological inactivation experiments also implicate the PL as an important structure in cocaine seeking (McFarland and Kalivas, 2001; Capriles et al., 2003; McLaughlin and See, 2003; McFarland et al., 2004; Peters et al., 2009). Congruent with previous literature our data suggest that PL ensembles play an important role in drug-related learning and expression of cocaine seeking. PL activity increased (as measured by β-gal expression) following exposure to the cocaine associated context after the acquisition of cocaine SA. Ablating these β-gal expressing neurons with Daun02 decreased cocaine SA both after acquisition and once rats were well-trained.

One limitation of our study is that we did not directly test whether ablating a similar number of neurons in the PL that are not associated with the cocaine environment or cues would produce the same result (i.e., neurons activated by a novel context control, or by a non-drug reward). While not directly tested here, previous reports using the Daun02 inactivation procedure have shown that ablating a small number of neurons not associated with the relevant context and cues does not influence drug seeking behavior (Suto et al., 2016; Warren et al., 2016, 2019; Kane et al., 2021). Therefore, it is unlikely that the decrease in cocaine seeking demonstrated in our studies is due to the non-specific inactivation of neurons in the PL. It is also important to note that our induction session is carried out under non-reinforcing conditions, therefore it is likely that some level of behavioral extinction occurs on induction day, leaving the possibility that we have also inactivated this fledgling extinction ensemble. Since our experiments are designed to test the rats’ ability to recall cocaine seeking, it is important that the rat is not being actively reinforced during the induction and final test session, as reinforcement could lead to new learning and recruitment of a new neuronal ensemble. To minimize the level of extinction learning occurring on induction and test day, we have limited these tests to 30 min. Furthermore, if our manipulation only targeted the extinction ensemble, we would expect to see an increase in active lever responding during the final test rather than a decrease, as this has been previously demonstrated (Warren et al., 2019). Therefore, we expect that any potential extinction learning occurring on induction day has a minimal influence on our findings. Furthermore, we did not test whether these neurons undergo synaptogenesis to form new connections during initial acquisition, thereby forming a novel ensemble, or whether these neurons are already connected prior to cocaine self-administration. Current IEG-based methods preclude the possibility of labeling the neuronal ensemble *a priori*, which limits their utility in identifying neurons that are destined to be part of a given ensemble. Nevertheless, understanding whether individual neurons are recruited into novel ensembles or whether extant proto-ensembles are adapted to control behavior is a critical question for future research.

Our experiments add an important layer of nuance to this literature due to the specificity of our technique. Only neurons active during the induction session are inactivated in our procedure. This is an important distinction to make, as previously held dichotomous thinking about mPFC functionality has led to conflicting evidence. For example, previous hypotheses that the PL drives cocaine seeking while the IL inhibits cocaine seeking (Rhodes and Killcross, 2004, 2007; Peters et al., 2009) conflict with evidence that ensembles in the IL not only mediate extinction but also SA of both heroin and cocaine (Koya et al., 2009a; Bossert et al., 2011; Warren et al., 2019; Kane et al., 2021). Here we find that only the minority of neurons that were activated because of exposure to the cocaine context and associated cues were required for cocaine seeking.

## Conclusion

Our findings further implicate the PL broadly in cocaine SA both after early learning and once rats are well-trained. The use of ensemble-specific manipulations in this study further highlights the importance of ensemble manipulations rather than region-wide investigation. It is important to note that while we did test at two distinct time points in the learning process, we do not know whether these are separate groups of neurons or an overlapping population.

## Data Availability Statement

The raw data supporting the conclusions of this article will be made available by the authors, without undue reservation.

## Ethics Statement

The animal study was reviewed and approved by University of Florida IACUC.

## Author Contributions

BW and BS designed the behavioral experiments. BW, CG, BS, BC, and SR performed surgeries or intracranial infusions on the rats and were responsible for collecting behavioral and histochemical data. BS and BW wrote the manuscript. All authors contributed to the article and approved the submitted version.

## Funding

BW was supported by a grant from the National Institute on Drug Abuse (NIDA: Grant R00DA042102), a NARSAD Young Investigator Grant from the Brain and Behavior Research Foundation, and support from The Center For Integrative Cardiovascular and Metabolic Disease at the University of Florida. Oxycodone was supplied by the NIH Drug Supply Program.
